# Supplementary material for: What Is Rural Adversity, How Does It Affect Wellbeing and What Are the Implications for Action?
Source: Int J Environ Res Public Health. 2020 Oct 1;17(19):7205. doi: 10.3390/ijerph17197205 (PMC7578975; doi:10.3390/ijerph17197205)
Supplement: Supplementary file 1 [file ijerph-17-07205-s001.pdf]

Supplementary Table 1 – Expert interdisciplinary group

Name	Position
Prof David Perkins (BA, PhD)	Director, Centre for Rural and Remote Mental Health, University of Newcastle, Orange NSW
Dr Hazel Dalton (BSc, PhD)	Research Leader, Centre for Rural and Remote Mental Health, University of Newcastle, Orange NSW
Prof Jane Farmer (BA, PhD)	Research Professor of Health & Social Innovation and Director, Social Research Innovation Institute, Swinburne University of Technology, Melbourne Vic
A/Prof Amir Aryani (PhD)	Head of Social Data Analytics (SoDA) lab, Social Research Innovation Institute, Swinburne University of Technology, Melbourne Vic
Prof Luis Salvador-Carulla (MD, PhD)	Head, Centre for Mental Health Research, Research School of Population Health, the Australian National University, Canberra ACT
Dr Nasser Bagheri (M.PH, PhD)	Co-director of the Visual and Decision Analysis (VIDEA) Lab, Centre for Mental Health Research, Research School of Population Health, the Australian National University, Canberra ACT
Prof Brian Kelly (BM, PhD, FRANZCP, FACPM)	Head of School of Medicine and Public Health; Co-Director, Priority Research Centre for Brain and Mental Health, and Director, Centre for Resources, Health and Safety, University of Newcastle, Newcastle NSW
A/Prof Georgina Luscombe (BSc, PhD)	Senior Lecturer in Rural Health, Medical Statistics, Medicine, School of Rural Health, University of Sydney, Orange NSW
A/Prof Mathew Coleman (MBBS, FRANZCP)	Associate Professor in Rural and Remote Mental Health, Rural Clinical School of Western Australia, University of Western Australia and Consultant psychiatrist, WA Country Health Service, Albany WA; Commissioner, Australian Mental Health Commission
A/Prof Nelly Oelke (BScN, MN, PhD, RN)	Associate Professor, School of Nursing, Faculty of Health and Social Development, University of British Columbia, and Scientific Director, Rural Coordination Centre of British Columbia, Okanagan, Canada
Dr Joanne Lawrence-Bourne (PhD)	Research Associate, Centre for Rural and Remote Mental Health, University of Newcastle, Orange NSW
